# A micropore array-based solid lift-off method for highly efficient and controllable cell alignment and spreading

**DOI:** 10.1038/s41378-020-00191-5

**Published:** 2020-09-07

**Authors:** Tingting Hun, Yaoping Liu, Yechang Guo, Yan Sun, Yubo Fan, Wei Wang

**Affiliations:** 1 Key Laboratory for Biomechanics and Mechanobiology of Ministry of Education, School of Biological Science and Medical Engineering, Beihang University, 100191 Beijing, China; 2 Institute of Microelectronics, Peking University, 100871 Beijing, China; 3 State Key Laboratory of Transducer Technology, Chinese Academy of Sciences, 200050 Shanghai, China; 4 Beijing Advanced Innovation Center for Biomedical Engineering, Beihang University, 100083 Beijing, China; 5 National Key Laboratory of Science and Technology on Micro/Nano Fabrication, 100871 Beijing, China; 6 Frontiers Science Center for Nano-optoelectronics, Peking University, 100871 Beijing, China

**Keywords:** Electrical and electronic engineering, Chemistry

## Abstract

Interpretation of cell–cell and cell-microenvironment interactions is critical for both advancing knowledge of basic biology and promoting applications of regenerative medicine. Cell patterning has been widely investigated in previous studies. However, the reported methods cannot simultaneously realize precise control of cell alignment and adhesion/spreading with a high efficiency at a high throughput. Here, a novel solid lift-off method with a micropore array as a shadow mask was proposed. Efficient and precise control of cell alignment and adhesion/spreading are simultaneously achieved via an ingeniously designed shadow mask, which contains large micropores (capture pores) in central areas and small micropores (spreading pores) in surrounding areas contributing to capture/alignment and adhesion/spreading control, respectively. The solid lift-off functions as follows: (1) protein micropattern generates through both the capture and spreading pores, (2) cell capture/alignment control is realized through the capture pores, and (3) cell adhesion/spreading is controlled through previously generated protein micropatterns after lift-off of the shadow mask. High-throughput (2.4–3.2 × 10^4^ cells/cm^2^) cell alignments were achieved with high efficiencies (86.2 ± 3.2%, 56.7 ± 9.4% and 51.1 ± 4.0% for single-cell, double-cell, and triple-cell alignments, respectively). Precise control of cell spreading and applications for regulating cell skeletons and cell–cell junctions were investigated and verified using murine skeletal muscle myoblasts. To the best of our knowledge, this is the first report to demonstrate highly efficient and controllable multicell alignment and adhesion/spreading simultaneously via a simple solid lift-off operation. This study successfully fills a gap in literatures and promotes the effective and reproducible application of cell patterning in the fields of both basic mechanism studies and applied medicine.

## Cell patterning: harnessing an ingeniously designed micropore array

A solid lift-off method with an ingeniously designed micropore array as a shadow mask enables the efficient and precise control of cell patterning. Cell patterning is an important strategy for both basic biological mechanism studies and applicable technology developments in regenerative medicine, such as tissue engineering. Here, a team led by Prof. Wei Wang from Peking University presents the first report of a simultaneous control of cell alignment and adhesion/spreading via an easy single-step lift-off operation. The key concept is that the micropore array contains the large pores in central areas and small pores in surrounding areas controlling cell capture and alignment, and cell adhesion and spreading, respectively. They demonstrated high-throughput, high-efficiency cell alignment along with a precise control of cell spreading.

## Introduction

Cell patterning is very useful to reveal the mechanisms of cell physiological processes, such as gene expression^1^, apoptosis^2^, differentiation^3^, and migration^4^. It is also widely applied in studies of drug discovery^5^, cancer invasion^6^, neuronal differentiation^7^, and organotypic tissue construction^8^. For example, to clarify stem cell microenvironments affecting both cell differentiation and self-renewal, controlling the relative location of cells can be used to manipulate cell–cell diffusible signaling or cell–cell junctions^9^. Another typical example is the use of cell patterning to construct precisely controlled tissue-engineered muscles for the treatment of skeletal muscle diseases, in which the controllable alignment and adhesion/spreading of myoblasts are particularly important for myotube formation^10,11^.

During the last two decades, many cell patterning methods have been developed. Cell patterning methods can be mainly classified into two types: active and passive approaches. The active approaches include dielectrophoresis (DEP)^12^, optoelectronic tweezers (OET)^13^, and magnetic^14^ and acoustical force^15,16^-based control which are often dependent on the specific equipment. Nevertheless, the passive approaches have attracted much attention in biological practices because of their simple operation, which mainly include microwell-assisted cell capture and confinement^17–21^ and protein micropattern-controlled cell alignment and spreading^22–29^. For evaluation of cell patterning techniques, the following points should be considered: (1) patterning (including simultaneous control of alignment and adhesion/spreading) efficiency and throughput with an easy operation, (2) controllability at the single-cell level, and (3) guarantee of cell viability, cell spreading, and proliferation ability after patterning. A comparison of the previously reported passive approaches with regard to the three described characteristics is provided in Table 1.

**Table 1:** A comparison of typical cell patterning methods

Ref. No.	Strategy design	Throughput^a^	Cell capture/patterning	Control of multicell alignment	Control of cell spreading	Comments
^18^	Microwell (single small well)	1800 wells/mm^2^	Single cell: 92%	No	No	Easy operation and high throughput; Influence on cell spreading and limitation to only short-term study
^19^	Microwell (combination of small and large wells)	~ 5.7 wells/mm^2^	Single cell: 77%	No	Yes	Feasibility for long-term study; Labor-intensive/time-consuming operation and low throughput
^20^	Microwell + Micropattern	~25 patterns/mm^2^	Single cell: 73.7 ± 8.1%	No	Yes	Feasibility for long-term study; Labor-intensive/time-consuming operation and low throughput
^26^	Micropattern (composite protein micropatterns via microcontact printing)	~240 patterns/mm^2^	Single-cell: 36.5 ± 3.3% Double-cell: 32.1 ± 1.9% Triple-cell: 24.2 ± 2.8%	Yes	Yes	Easy operation; Low overall efficiency of cell capture/alignment and adhesion/spreading
^27^	Micropattern (protein micropatterns via inkjet printing)	~1 pattern/6.5 mm^2^	Cell group	No	Yes	Low cost and easy operation; Limitation to large feature size and unsuitability for single-cell patterning
^35^	Micropattern (protein micropatterns via UV controlled crosslinking)	~6 patterns/mm^2^	Cell group	No	No	Easy operation and strong stability; Limitation to large feature size and unsuitability for single-cell patterning
^25^	Micropattern (protein micropatterns via electrochemically induced selective absorption)	~3 patterns/mm	Cell group	No	Yes	Easy operation; Limitation to large feature size and unsuitability for single-cell patterning
^29^	Micropattern (protein micropatterns via electrochemically induced selective absorption)	~7 patterns/mm	Cell group	No	Yes	Easy operation; Limitation to large feature size and unsuitability for single-cell patterning
^21^	Micropattern (protein micropatterns via solid lift-off)	~210 patterns/mm^2^	Single cell: ~30%^b^	No	No	Easy operation, high throughput and feasibility of single-cell patterning; Challenging trade off of single-cell capture efficiency and spreading requirement
^23^	Micropattern (protein micropatterns via solid lift-off)	~500 patterns/mm^2^	Single cell: 53%	No	No	Easy operation, high throughput and feasibility of single-cell patterning; Challenging trade off of single-cell capture efficiency and spreading requirement
^37^	Micropattern (protein micropatterns via solid lift-off)	~25 patterns/mm^2^	Cell group	No	No	Easy operation; Limitation to large feature size and unsuitability for single-cell patterning
This study	Solid lift-off (simultaneous control of protein micropatterns generation and cell alignment/adhesion/spreading)	~320 patterns/mm^2^	Single-cell: 86.2 ± 3.2% Double-cell: 56.7 ± 9.4% Triple-cell: 51.1 ± 4.0%	Yes	Yes	Easy operation, high throughput, high overall cell patterning efficiencies, supports for long-term functional study; high cost of shadow mask fabrication

^a^A conversion of throughput to quantity of microwells or micropatterns/mm^2^ for parallel comparison

^b^Calculated value according to the figures in original references

Microwell-assisted methods (driven by gravity or hydrodynamics) are attractive strategies owing to their simplicity and low expertise requirements. However, a conflict exists between the single-cell capture efficiency ensuring and cell spreading area control when determining the optimal microwell size. A relatively small well size is necessary to ensure a high-efficiency capture of single cell^17^, but it limits the long-term analysis owing to the inefficient support and spreading area for cell growth^18^. To address these issues, Lin et al.^19^ realized further cell spreading and growth by transferring the captured cells from small wells to large wells; however, the transfer operation was complicated (requiring alignment), readily caused cell loss, and influenced cell viability. Later, Tu et al.^20^ designed a combination of the microwell array and micropattern array, first achieving single-cell capture in the microwell and then transferring the captured cells to micropatterns for sufficient spreading. However, a precise alignment system was required in the chip preparation and experimental setup, and thus, this method is labor intensive, time consuming, and unfriendly for wide biomedical applications.

Protein micropattern-controlled methods have also emerged as useful tools for cell patterning, with various geometries to accurately control both the location/adhesion of cells and the spreading shape/area for long-term functional studies. Recently, several methods for protein micropattern preparation have been reported, including microcontact printing^2,22,26,28,30–33^, inkjet printing^27,34^, mask-based UV controlled protein crosslinking^35,36^, electrochemically induced spatially selective protein absorption and patterning^25,29^, and solid lift-off methods^23,37,38^. Notably, almost all of these methods have been limited to a large feature size, owing to the limitation of control precision. These large-sized protein micropatterns have been successfully utilized for cell migration^28^, cytoskeleton alignments^39^, and myotube formation^33^ at the cell group level. However, they were unsuitable for preparing micropatterns of a size comparable to the diameter of an individual cell and correspondingly fulfilling the study requiring controllable spatial distribution at the single-cell level. Some attempts to utilize microcontact and inkjet printing to realize small sizes have suffered defects, resulting from stamp deformation/collapse^40,41^ and poor resolution^27^, respectively. Recently, small micropatterns of 15 μm and 20 μm were achieved by Lee et al.^23^ and Wu et al.^21^ via lift-off. However, the reported lift-off strategies cannot realize a small space between the patterned adjacent cells, limited by the fabrication capacity for shadow masks and corresponding protein micropatterns. The control of cell alignment and adhesion/spreading was still totally dependent on the protein adhesion confinement and thus suffered low efficiency. In short, all the protein micropattern controlling adhesion-based methods must overcome the problem of low overall patterning efficiency, which is caused by the difficult trade-off of the pattern size, constrained by the conflict between single-cell capture efficiency and spreading requirement/control including the area and shape.

**Fig. 1:**
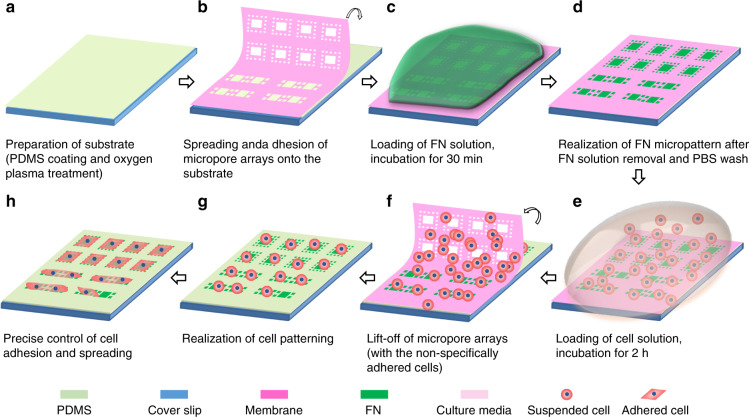
Schematic illustration of protein micropattern generation and cell alignment and adhesion/spreading control via the proposed solid lift-off method. **a**−**d** protein micropattern generation in both the capture and spreading pores. **e**−**f** cell capture/alignment control via the capture pores. **g**−**h** cell adhesion/spreading control through previously generated protein micropatterns after lift-off of the shadow mask

In our previous study, a composite pattern (large patterns in the central areas for cell location/alignment and small patterns in the surrounding areas for cell adhesion/spreading control) prepared using the microcontact printing technique was applied for controllable single-cell (36.5 ± 3.3%), double-cell (32.1 ± 1.9%), and triple-cell (24.2 ± 2.8%) alignments^26^. The implementation of composite patterns avoided the aforementioned trade-off problem in pattern size. However, the efficiency was still limited with regard to cell location/alignment simply based on the protein micropattern confining, although the cell seeding density was carefully optimized. In this study, a single-step operation, solid lift-off, was proposed for high-efficiency and high-throughput cell patterning, free from the aforementioned trade-off problem. A flexible micropore array acted as the shadow mask in the proposed solid lift-off method. The key is an ingenious design of the shadow mask, which contains large micropores (capture pores) in the central areas and small micropores (spreading pores) in the surrounding areas, controlling capture/alignment and adhesion/spreading, respectively. This solid lift-off fulfilled simultaneous control of cell alignment and adhesion/spreading as follows: (1) protein micropattern generation through both the capture and spreading pores, (2) cell capture/alignment control through the capture pores, and (3) cell adhesion/spreading control through previously generated protein micropatterns after lift-off of the shadow mask. The above flow paths were realized via a single-step lift-off with exchange of the protein solution and the cell solution. High-throughput and high-efficiency protein micropatterning (patterning on flat and curved substrates) and cell alignment and adhesion/spreading control were successfully obtained, as shown in Fig. 1. Additionally, benefiting from the size precision in the preparation of Parylene C micropore arrays established in our previous report^42^, a small space (5 μm) between the patterned adjacent cells could be obtained, thus allowing the designable control of cell–cell junctions and cytoskeleton distribution, which was investigated using C2C12 (murine skeletal muscle myoblast) cells.

## Results and discussions

### Solid lift-off method

**Fig. 2:**
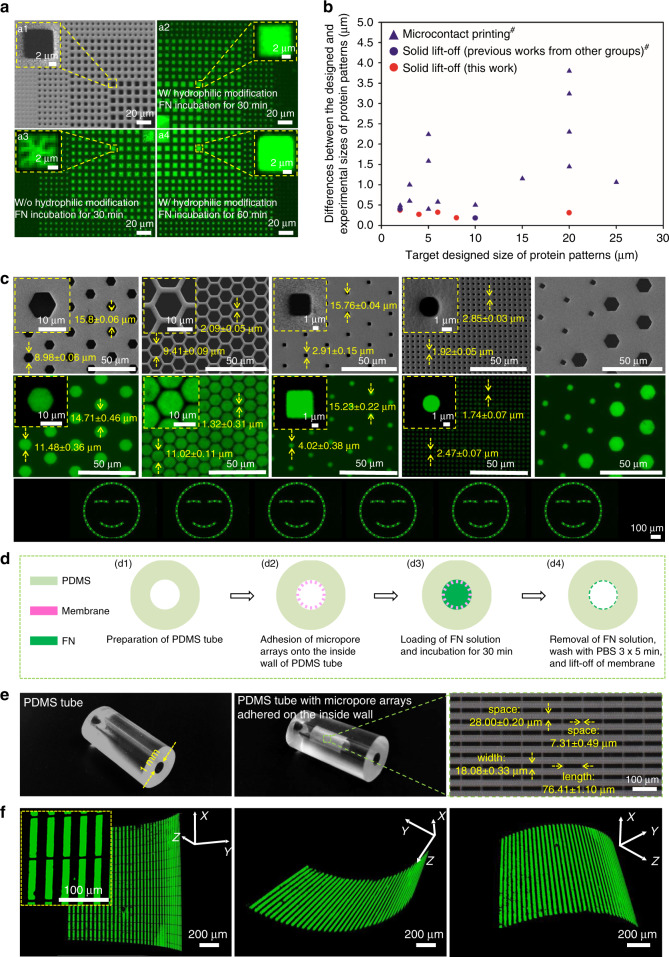
Protein (Alexa Fluor 488-conjugated FN) patterning with the solid lift-off method. **a** Protein micropatterns obtained with different surface treatments of substrates and incubation time lengths of FN solution. **b** Differences between the designed and experimentally obtained sizes of protein micropatterns by the present solid lift-off method and previously reported techniques^23,26,49–53^. **c** Typical scanning electron microscopy (SEM) images of the Parylene C micropore arrays (shadow masks) and fluorescence images of the corresponding protein micropatterns of various shapes and sizes on flat substrates. **d** Schematic illustration of protein micropattern preparation on curved substrates via the solid lift-off method. **e** PDMS tubes without and with micropore arrays adhered on the inside walls, and the SEM image of the used micropore arrays. **f** Typical confocal images of the achieved protein micropatterns on the curved substrates (i.e., the inner wall surface of PDMS tubes with radius of curvature at 0.5 mm), in views of different angles. ^#^These values were calculated according to the figures in the original references

To optimize the proposed solid lift-off protocol, two aspects including the surface treatment of substrates and time length of protein (taking fibronectin (FN) as an example in this work) incubation, were carefully considered. FN patterns with good uniformity were achieved on the substrate with hydrophilic modification before FN incubation for 30 min, as displayed in Fig. 2a2. However, some defects arose (discontinuity and nonuniformity) in the obtained FN patterns after FN incubation for 30 min, as shown in Fig. 2a3, if the surface of the substrate was not treated with oxygen plasma for hydrophilic modification, which would interfere with the cell adhesion and spreading in cellular studies. Moreover, when the FN incubation time increased from 30 to 60 min, the FN expanded more out of the pore area (on the shadow mask), resulting in a larger deviation in size between the practically obtained and designed patterns, as shown in Fig. 2a4. Thus, the optimized performing conditions for high-quality FN patterns comprised hydrophilic modification of substrates prior to adhesion of the Parylene C micropore arrays and incubation of the FN solution for 30 min. With the optimized protocol, FN patterns of various shapes and sizes could be well prepared (Fig. 2c). All the subsequently presented FN patterns were prepared using the optimized condition unless otherwise stated. Additionally, the differences between the designed and practically obtained sizes of protein micropatterns (*d*) prepared using the present solid lift-off and previously reported methods were compared, as shown in Fig. 2b. The data in Fig. 2b indicate that the proposed solid lift-off method is more reliable in achieving a small size of protein patterning with a high size precision, with *d* < 0.5 μm (red dots in Fig. 2b), i.e., only a small percentage (6.9 ± 6.1%) change was observed in the experimental values compared to the designed ones.

However, the microcontact printing technique prepares small protein micropatterns with a larger value of *d* (varying from 0.35 to 3.8 μm, blue triangles in Fig. 2b), i.e., a larger percentage (19.4 ± 11.8%) change relative to the designed value. It is conceivable that the size of the prepared patterns in the microcontact printing process is easily influenced by the applied pressure during the transfer of FN from stamps to substrates, as shown in Supplementary Fig. S1, which often varies among operators. Therefore, the differences between the experimental and designed values were large. In contrast, the proposed solid lift-off method is free of any extra pressure loading and thus more robust and stable, supporting its wide applications, particularly in fields with high requirements for size precision.

### Protein patterning on flat and curved substrates

As shown in Fig. 2c, FN patterns of various sizes and shapes on flat substrates were successfully prepared. The typical sizes/arrangements generated in this study included small diameter/small space (2.47 ± 0.07 μm/1.74 ± 0.07 μm), small diameter/large space (4.02 ± 0.38 μm/15.23 ± 0.22 μm), large diameter/small space (11.02 ± 0.11 μm/1.32 ± 0.31 μm), and large diameter/large space (11.48 ± 0.36 μm/14.71 ± 0.46 μm). The shapes generated in this study included uniformly distributed square or hexagon arrays, a combination of squares and hexagons, and arbitrary shapes (e.g., the pattern of a smile, ). However, some of the above distribution types (e.g., large diameter/small space and small diameter/large or small space) of protein micropatterns were unachievable by the previously reported methods. The failures resulted from collapses and deformations of PDMS microstructures with a high width/height ratio and a high height/width ratio, respectively, schematically shown in Supplementary Fig. S1 for the microcontact printing method, and poor precision in the fabrication capacity for inkjet printing and UV-induced crosslinking.

In our present solid lift-off technique, the success in achieving various arrangements of protein micropatterns with a high size precision, even for a small feature size (<2 μm), is attributed to the spatially confining protein transfer process (i.e., protein molecules contacted substrates only after traveling through micropores). Further studies of the detailed mechanism to illustrate how the protein molecules reach the substrate through micropores without extra driving force are ongoing. Moreover, protein micropatterns on curved substrates, e.g., the inner wall surface of PDMS tubes with various radiuses of curvature (0.5–3 mm), were also successfully obtained, benefiting from the flexibility of Parylene C micropore arrays (compatibility of the tube shadow mask). The typical fluorescence images of the obtained FN patterns on curved substrates with various radius of curvatures (*r)* are displayed in Fig. 2f and Supplementary Fig. S2a-b, with views of different angles. The patterning capacity on curved substrates will facilitate the promising applications of this method in organ-on-chips and tissue engineering, which involve complicated 3D structure constructions, particularly for ex vivo and in vivo studies.

### High-throughput and high-efficiency cell patterning

**Fig. 3:**
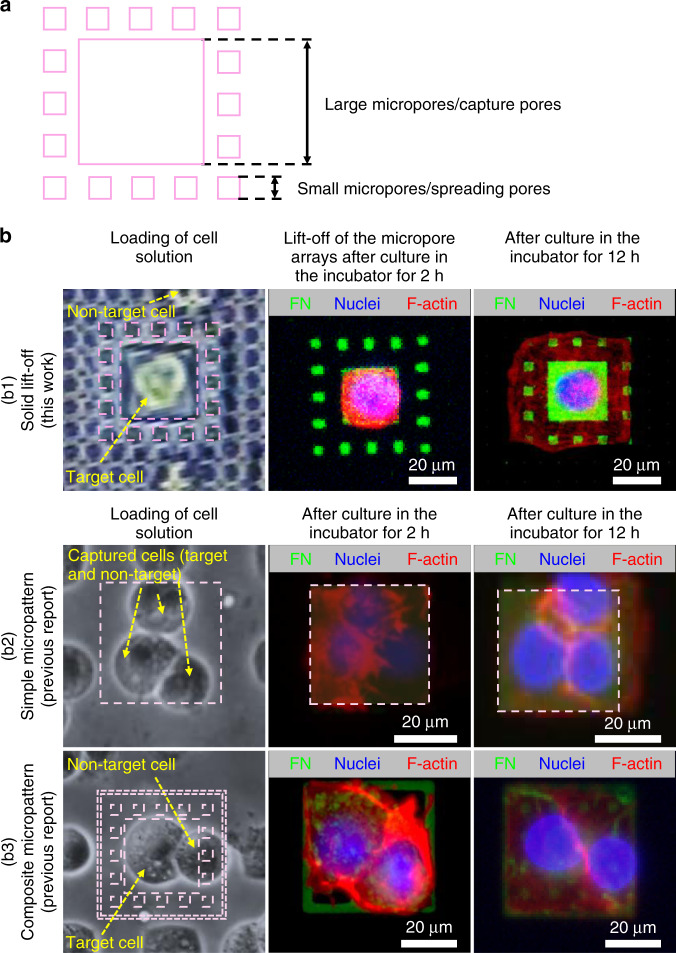
Principle of the shadow mask design via solid lift-off method and cell patterning via both the solid lift-off and microcontact-printing method. **a** Composite confining structures of the Parylene C micropore arrays. **b** Illustration of principles for cell patterning and typical images of FN micropatterns (green) and patterned cells with staining of nuclei (blue) and F-actin (red) generated by the present solid lift-off method (b1) and the previously reported protein micropattern-controlled method (e.g., microcontact printing^26^) (b2–b3)

**Fig. 4:**
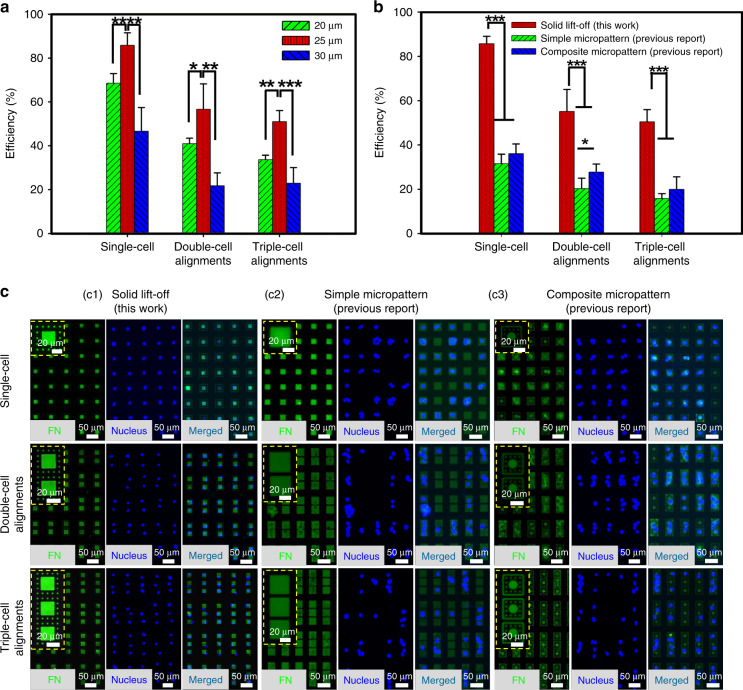
Results of cell patterning with the solid lift-off method. **a** Efficiencies of single-cell, double-cell, and triple-cell alignments via the solid lift-off method with shadow masks of different capture pore sizes (*n* = 3). **b** Comparison of the efficiencies for single-cell, double-cell, and triple-cell alignments via the present solid lift-off method (*n* = 3) and previously reported protein micropattern-controlled methods (microcontact printing^26^). **c** Typical images of FN micropatterns (green) and patterned cells (blue and merged) generated by the present solid lift-off method (c1) and previously reported protein micropattern-controlled methods (microcontact printing^26^) (c2–c3)

The results of cell patterning are shown in Figs. 3, 4. Previously, we confirmed the high efficiency of the solid lift-off method in achieving high-throughput single-cell capture with a simple shadow mask^43^. In this work, improvements have been made to realize multicell alignments along with simultaneous control of cell adhesion and spreading via an ingeniously designed shadow mask. The design philosophy of the composite shadow mask in the present solid lift-off method comprises the large micropores (capture pores) in the central areas controlling cell capture/alignments and the small micropores (spreading pores) in the surrounding areas controlling cell adhesion/spreading. The size of the capture pores strongly influences the efficiency of cell alignments, as shown in Fig. 3a. The reasons why the present solid lift-off method can achieve such high efficiencies in high-throughput cell patterning are discussed and schematically illustrated in Fig. 3b. In the process of cell loading and incubation (2 h), only a single cell could be captured in a capture pore, although some cells nonspecifically adhered on the membrane (shadow mask) in the areas beyond the capture pores. Subsequently, the nonspecifically adhered cells were removed together with the lift-off of the shadow mask (Parylene C micropore arrays), leaving only cells in the capture pores to adhere and spread (12 h) in the patterning areas confined by the spreading pores (Fig. 3b1). The removal of nonspecifically adhered cells is a critical factor to obtain high-efficiency cell alignments at a high throughput. It is easy to be thought that if the cells in the micropores adhered on the wall of micropores, the cell alignment efficiency would be impacted when the mask was lifted off from the substrate. Nevertheless, this impact has been minimized via careful experimental optimizations with separate investigations of incubation duration lengths after the FN protein solution and cell solution loading for the high-efficiency protein and cell patterning.

In contrast, in the other protein micropattern-based methods, to meet the requirement of cell spreading, the size of the pattern was always larger than the diameter of an individual cell in suspension status (a result of the aforementioned trade-off). Therefore, two or more cells were often captured on protein micropatterns (Fig. 3b2, b3), without means to remove the undesired cells, resulting in poor controllability and a low efficiency of cell alignment. To the best of our knowledge, this is the first report to simultaneously establish the precise control of cell alignment and adhesion/spreading with a high efficiency at a high throughput through only a single-step operation, i.e., the developed solid lift-off method.

For optimization, different sizes of capture pores (20, 25, and 30 μm) were thus first investigated. As shown in Fig. 4a, a 25 μm capture pore presents the highest efficiencies for single-cell, double-cell, and triple-cell alignments of 86.2 ± 3.2%, 56.7 ± 9.4% and 51.1 ± 4.0%, respectively, compared to those of 20 μm (68.5 ± 3.7%, 41.0 ± 1.7%, and 33.7 ± 1.6%, respectively), and 30 μm (46.7 ± 9.1%, 21.8 ± 4.2%, and 22.8 ± 5.8%, respectively). All subsequent cell patterning results shown in this study were obtained with a capture pore size of 25 μm, unless otherwise stated. The confinement area for cell spreading (the total area spanned by capture pores and spreading pores) was 39 μm × 39 μm. The pitches were 69 μm, 44 μm, and 44 μm (i.e., spaces were 30, 5, and 5 μm) for single-cell, double-cell, and triple-cell alignments, respectively. Cell patterning on curved substrates (the inner wall surface of PDMS tubes with various radius of curvature) was also investigated, and the preliminary results are shown in Supplementary Fig. S2. In addition to the patterning of the same typed cells, the proposed solid lift-off method in this paper is also applicable to the patterning of different typed cells. The patterning of different typed cells is feasible referring to the previously reported multistep solid lift-off process with multilayer Parylene C micropore arrays as shadow masks. Taking the patterning of two typed cells as an example, the schematic illustration is shown in Supplementary Fig. S3. The previously reported results demonstrated that the cells were of high viability after multiple solid lift-off operations^44^^,45^. Therefore, the integration of our novel design for simultaneous control of cell alignment and adhesion/spreading and the multiple solid lift-off processes shows promise for fulfilling the patterning of multiple typed cells, and be of great potential and broad interests for applications in various fields including regenerative medicine and tissue engineering.

In addition, the cell patterning efficiencies obtained from the previous methods (e.g., microcontact printing), which controlled cell alignment totally depending on the protein adhesion confinement, were experimentally compared in parallel, as shown in Fig. 4b. Typical images of patterned cells are displayed in Fig. 4c. The achieved efficiencies with the present solid lift-off method are significantly higher than those of the protein micropattern-based methods. For single-cell alignment, the increase in proportions are 153.52% and 131.16% compared to the previous methods, simple and composite protein micropatterns^26^, respectively. The corresponding values for double-cell and triple-cell alignments are 141.27% and 76.6% and 250% and 111.15%, respectively.

### Application in the functional study of cell skeleton distribution and cell–cell junctions

**Fig. 5:**
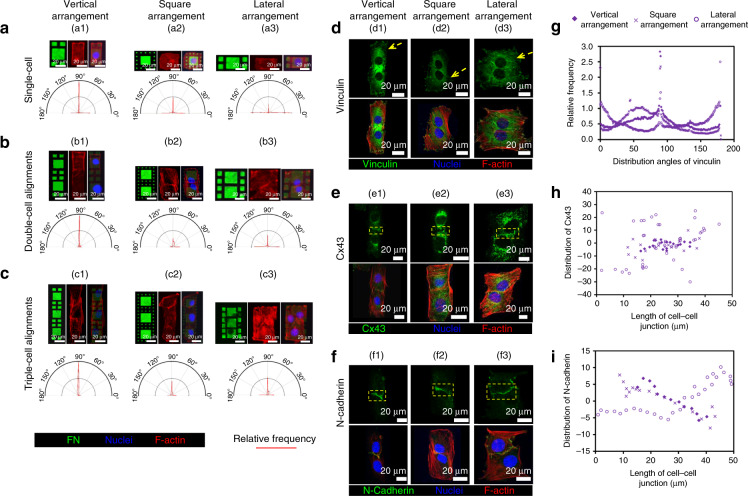
Results of F-actin alignment and distributions of three typical proteins in the cell–cell junction areas. Typical immunofluorescence images of single-cell (**a**), double-cell (**b**), and triple-cell (**c**) alignments on micropatterns with different arrangements/distributions (vertical, square, and lateral) and the extracted distributions of F-actin alignments obtained via MATLAB processing. Immunofluorescence images of vinculin (**d**), Cx43 (**e**), and N-cadherin (**f**) (double-cell alignments in vertical, square, and lateral arrangements). Distributions of vinculin (**g**), Cx43 (**h**), and N-cadherin (**i**) in the cell–cell junction areas, analyzed using ImageJ and MATLAB processing

The cytoskeleton (F-actin) alignment and distributions of three typical proteins (vinculin, Cx43, and N-cadherin) in the cell–cell junction areas were analyzed with murine skeletal muscle myoblasts (C2C12), as shown in Fig. 5. The orientation distributions of F-actin filaments are shown in Fig. 5a–c, including three different types of cell alignments, in vertical (a1–c1), square (a2–c2), and lateral (a3–c3) arrangements. From a previous report, the actin cytoskeleton will be strongly reorganized before myoblasts fusing and differentiating into myotubes, where the actin filaments tend to organize into a dense actin wall structure that parallels and extends the length of the plasma membrane of the aligned cells^46^. Here the consistent phenomena were observed. For single-cell patterning, in the type of vertical arrangement (Fig. 5a1), most of the F-actin filaments were distributed along the direction of 90^o^, i.e., along the long axis of the micropatterns (cells). In contrast, in the type of lateral arrangements, i.e., 90^o^ rotation from the vertical arrangement (Fig. 5a3), the F-actin filaments were mainly distributed along the directions of 0^o^ and 180^o^, i.e., 90^o^ rotation clockwise or counterclockwise rotation from that of the vertical arrangement (direction of 90^o^). In the type of square arrangement (Fig. 5a2), the F-actin filaments were randomly distributed along directions of 0^o^, 90^o^, and 180^o^ (i.e., where the plasma membrane boundary of patterned cells is distributed). Here one point needs noting that the “random” in this work means “the random percentages of F-actin distributions among directions of 0^o^, 90^o^ and 180^o^”, rather than “the F-actin distributions are in any angle along the circumferential direction”. In the cases of multicell patterning, in the vertical arrangement (Fig. 5a1–c1), the F-actin filaments were more significantly distributed along the direction of 90^o^, i.e., along the long axis of the entire alignment of micropatterns (cells), as expected. In contrast, in the other two groups (square and lateral arrangements in Fig. 5a2–c2 and a3–c3, respectively), the distributions of F-actin filaments became random. The distribution of the cytoskeleton (F-actin filament as a typical object) is considered important for cell–cell interaction and cell–cell junction formation. To verify this, distributions of three typical proteins in cell–cell junction areas were analyzed, as shown in Fig. 5d−f.

Vinculin, a membrane-cytoskeleton protein in focal adhesion plaques, participates in linkage of integrin adhesion molecules to the F-actin filament and is associated with cell–cell junctions by anchoring F-actin to the membrane. Therefore, the distribution of vinculin was predicted to follow that of F-actin, which was proved by the experimental results. In the vertical arrangement (Fig. 5d1), the distribution was focused in the direction of 90 ^o^, i.e., along the long axis of the entire arrangement of patterns/cells. In contrast, random distributions were observed in the other two control groups (square and lateral arrangements in Fig. 5d2, d3, respectively).

Cx43 and N-cadherin, which are involved in the formation of junctions to bind cells with each other, were also analyzed. As shown in Fig. 5e1, h and Fig. 5f1, i, the distributions of Cx43 and N-cadherin molecules were both focused in the direction along the main axis of cell–cell junctions (the borders of adjacent cells) in the vertical arrangement. In contrast, in the square and lateral arrangements, the distributions of both Cx43 and N-cadherin molecules were rambling (Fig. 5e2–3, h, and Fig. 5f2–3, i, respectively). The focused distributions of Cx43 and N-cadherin along the cell–cell border and the distributions of vinculin and F-actin along the long axis of the entire arrangement of patterns revealed that C2C12 cells preferred to form cell–cell junctions in the end-to-end way, i.e., vertically, rather than laterally or at right angles, which is consistent with a previous report^47^.

The precise control of cell alignments achieved by only a single-step solid lift-off operation realizes the ability to regulate the distribution of the cytoskeleton and cell–cell junctions, which are critical factors affecting subsequent signaling pathways involved in cell–cell interactions, differentiation, and further development into an organ or organism. The promising potential of the developed simple but effective method will facilitate its extensive utilization in regenerative medicine and tissue engineering for basic mechanism studies as well as practical applications.

## Conclusions

In this study, a novel solid lift-off method using a microfabricated micropore array as a shadow mask was successfully developed to obtain high-throughput protein patterning with high precision and cell patterning (alignment/adhesion/spreading) with high efficiency. Benefiting from the advantages of microfabrication, the shape and size of the micropore arrays, and thus the resulting protein micropatterns, could be feasibly designed and precisely controlled. Protein micropatterns with various shapes and arrangements (sizes and spaces) were suitably prepared, including dimensions approximating an individual cell, which were unachievable using previously reported protein micropattern-based methods. In addition, the flexibility of Parylene C micropore arrays allowed protein and cell patterning on curved substrates. Notably, this solid lift-off method successfully fills a gap in the literature as the first report to demonstrate the precise control of cell alignment and adhesion/spreading simultaneously at a high throughput with high efficiencies. Overall, the high performances could be attributed to the ingenious design of the shadow mask in the solid lift-off process, which consists of large central micropores (capture pores) for cell capture/alignment and small surrounding micropores (spreading pores) for protein micropattern generation and cell adhesion/spreading control. In our previous reports^26^, a similar composite protein micropattern was proposed to control cell alignment and spreading free from the aforementioned trade-off problem. However, the composite protein micropattern was prepared with the conventional microcontact printing and thus cannot still overcome the problem of low efficiency because its control of cell alignment was totally dependent on the protein adhesion confinement. Nevertheless, this work totally avoids the aforementioned trade-off problem and shows high efficiency in simultaneous control of cell alignment and adhesion/spreading at a high throughput through a novel and simple solid lift-off method. Furthermore, this method could be successfully applied to control cell alignment and assess its influence on cytoskeleton arrangement and cell–cell junction formation, which can further fulfill the generation of complex tissue morphologies, such as spatially organized cell cultures and organoid development. The microfabrication and operation of the developed solid lift-off method are straightforward while retaining a high throughput and a high efficiency for protein and cell patterning. Overall, this study will bring extensive benefits for the efficient and reproducible application of cell patterning methodologies in the fields of both basic mechanism research and applied medicine, including organ-on-a chip and tissue engineering.

## Materials and methods

### Preparation of substrates

For the preparation of flat substrates, a cover slip of 25 mm diameter was ultrasonically cleaned sequentially with acetone, 75% ethanol, and deionized water (DI water) for 5 min each. After heat-drying, the cleaned coverslip was spin-coated with polydimethylsiloxane (PDMS) (Sylgard 184, Dow Corning, Armonk, NY, USA, 10:1 ratio of base to curing agent) at 1000 rpm for 10 s and 4000 rpm for 60 s to form a 15 μm-thick film, followed by curing at 65 °C for 12 h on a heating plate.

For preparation of the curved substrates (inner wall surfaces of the PDMS tubes), commercially available smooth glass rods of different diameters, ranging from 1 to 6 mm, were used as the templates. The glass rods were first cleaned following the same procedure as for the cover slip. The PDMS prepolymer was then poured onto the glass rods and cured at 65 °C for 12 h on a heating plate. Finally, sonication in acetone for 20 min was performed to obtain the release of the tube after PDMS swelling, in accordance with a previously reported method^48^. The released PDMS tubes were rinsed three times with 1X phosphate buffered saline solution (PBS) (pH 7.2–7.4, 10010023, Gibco/Thermo Fisher, Waltham, MA, USA) and then immersed in PBS until use for protein patterning.

### Design and fabrication of Parylene C micropore arrays

Different Parylene C micropore arrays were designed for protein and cell patterning; more detailed information regarding the shape and size is shown in Figs. 2, 3. The Parylene C micropore arrays used in the present study were prepared via a molding technique, which was previously reported by our group^42^.

### Preparation of cells

Murine skeletal muscle myoblast (C2C12 cell), purchased from the American Type Culture Collection (CRL-1722, ATCC, Manassas, VA, USA), was used as a model cell in this study. C2C12 cells were cultured in the high-glucose Dulbecco’s modified Eagle medium (DMEM-high glucose, Corning) with 10% (v/v) fetal bovine serum (HyClone, Smithfield, Australia). When the confluency reached 80–90%, the cells were trypsinized from the flask, centrifuged at 1000 rpm for 5 min, and then resuspended in DMEM at a concentration of 5 × 10^5^ cells/mL for use in cell patterning.

### Protein and cell patterning via the solid lift-off method

First, exposure of the top surface of PDMS-coated substrates (flat substrates) and the PDMS tubes (curved substrates) with the cross section facing the ultraviolet lamp in a UV/ozone cleaner (PSD Pro, Novascan, Milwaukee, WI, USA) for 15 min was performed for hydrophilic treatment, as shown in Fig. 1a, 2d1. As a parallel control, no hydrophilic treatment was also applied. Next, the deionized water (DI water)-wetted Parylene C micropore arrays were placed onto the top surface of the pretreated flat substrate (Fig. 1b) and the inside wall surface of the pretreated PDMS tube (curved substrate, Fig. 2d2) with tweezers. The Parylene micropore-arrayed membrane could naturally fit to the flat substrate and inside the wall surface of the tube because of its high flexibility. Third, after the residual water on the top surface of the micropore arrays was blow-dried with a rubber suction bulb, 200 μL FN solution (BD Biosciences, San Jose, CA, USA, diluted in sterilized DI water, at a concentration of 25 μg/mL) was dropped onto the Parylene C micropore arrays and incubated for 30 min at room temperature (27 °C) (Fig. 1c, Fig. 2d3) to ensure that sufficient FN molecules pass through the pores and form protein micropatterns on the substrate. The 30 min incubation was from experimental optimization. Then, the extra FN solution was removed, and the substrate (with Parylene C micropore arrays adhered on) was washed three times with 1X PBS to realize the FN pattern (Fig. 1d and 2d4). Subsequently, 200 μL C2C12 cell solution (5 × 10^5^ cells/mL) was dropped onto the Parylene C micropore arrays and incubated in the cell culture incubator for 2 h (Fig. 1e). The 2 h incubation was from experimental optimization. Following incubation, the Parylene C micropore arrays were lifted off from the substrate, dispersing the nonspecifically adhered cells (Fig. 1f) to obtain the designed cell alignments with the underlying protein micropatterns (Fig. 1g) and spread with precise control, as displayed in Fig. 1h. Alternatively, if only protein micropatterns were needed in practical applications, the Parylene C micropore arrays could be lifted off from the substrate after the PBS wash step.

### Immunofluorescence staining for the cytoskeleton and corresponding protein analysis

After culture for 12 h, the cells were washed with PBS (containing 1.0 mM CaCl_2_ and 0.5 mM MgCl_2_), fixed with 4% paraformaldehyde at room temperature for 15 min, and washed three times with PBS for 5 min each. Next, the cells were permeabilized with 0.1% Triton X-100 (Sigma-Aldrich, St. Louis, MO, USA) at 4 °C for 8 min, followed by washing with PBS three times for 5 min each. Cells were then blocked with 5% bovine serum albumin (Ruierxinde, China, diluted in PBS) through incubation at 37 °C for 1 h, incubated with an anti-FN antibody (1:200 dilution, rabbit-anti-mouse, Sigma), anti-connexin 43 (anti-Cx43) antibody (1:200 dilution, rabbit-anti-mouse, CST, Danvers, MA, USA), anti-N-cadherin antibody (1:200 dilution, rabbit-anti-mouse, CST), or anti-vinculin antibody (1:200 dilution, rabbit-anti-mouse, Sigma) in blocking buffer (1% bovine serum albumin) at 37 °C for 1 h, followed by washing three times with PBS for 5 min each. Subsequently, the cells were incubated with Alexa Fluor 488-conjugated secondary antibody (goat anti-rabbit, 1:100 dilution, Beijing Biosynthesis Biotechnology, Beijing, China), DAPI (1 μg/mL, Life Technologies, Thermo Fisher), and the cytoskeletal marker rhodamine-conjugated phalloidin (1:100 dilution, Sigma) in blocking buffer at room temperature for 1 h, followed by washing three times with PBS for 5 min each. Finally, the stained cells were mounted on glass slides using Prolong Gold Antifade (Life Technologies). All samples were imaged under a laser scanning confocal microscope (Leica Tcs SPE, Nussloch, Germany).

### Calculation of cell alignment efficiency

The efficiency of single-cell capture was calculated by dividing the number of protein micropatterns with a single-cell captured by the total number of protein micropatterns. The efficiencies of double-cell and triple-cell alignments were calculated following the above description, with the counting units as double and triple capture pores (cells), respectively. All data are presented as the mean ± standard deviation (SD). T-tests were used for the comparison of efficiency values from the present solid lift-off method and previously reported microcontact printing technique. The statistical significance is indicated with asterisks: *p < 0.05, **p < 0.01, and ***p < 0.001.

### Analysis of cytoskeleton alignments and protein distributions in cell–cell junctions

The images of immunofluorescently stained F-actin and vinculin (Fig. 5a) were processed using a home-programmed MATLAB processing. The vector orientations of F-actin filaments and angle of vinculin distribution were calculated based on the extracted pixel intensity gradients. In these analyses, the direction perpendicular to the long axis of the entire alignment of micropatterns was set as the angle of 0^o^. The images of Cx43 and N-cadherin were analyzed using the open source software ImageJ. The distributions of Cx43 and N-cadherin in the cell–cell junction areas were extracted.

## Supplmentary information

Supplmentary Information
