# Supplementary material for: A micropore array-based solid lift-off method for highly efficient and controllable cell alignment and spreading
Source: Microsyst Nanoeng. 2020 Sep 7;6:86. doi: 10.1038/s41378-020-00191-5 (PMC8433473; doi:10.1038/s41378-020-00191-5)
Supplement: Supplementary file 1 — Supplmentary Information [file 41378_2020_191_MOESM1_ESM.docx]

**A Micropore Array-based Solid Lift-Off Method for Highly Efficient and Controllable Cell Alignment and Spreading**

Tingting Hun †, Yaoping Liu †, Yechang Guo , Yan Sun ^*^, Yubo Fan ^*^and Wei Wang ^*^

Electronic Supplementary Information (ESI) available: Fig. S-1, Fig. S-2 and Fig. S-3. See DOI:


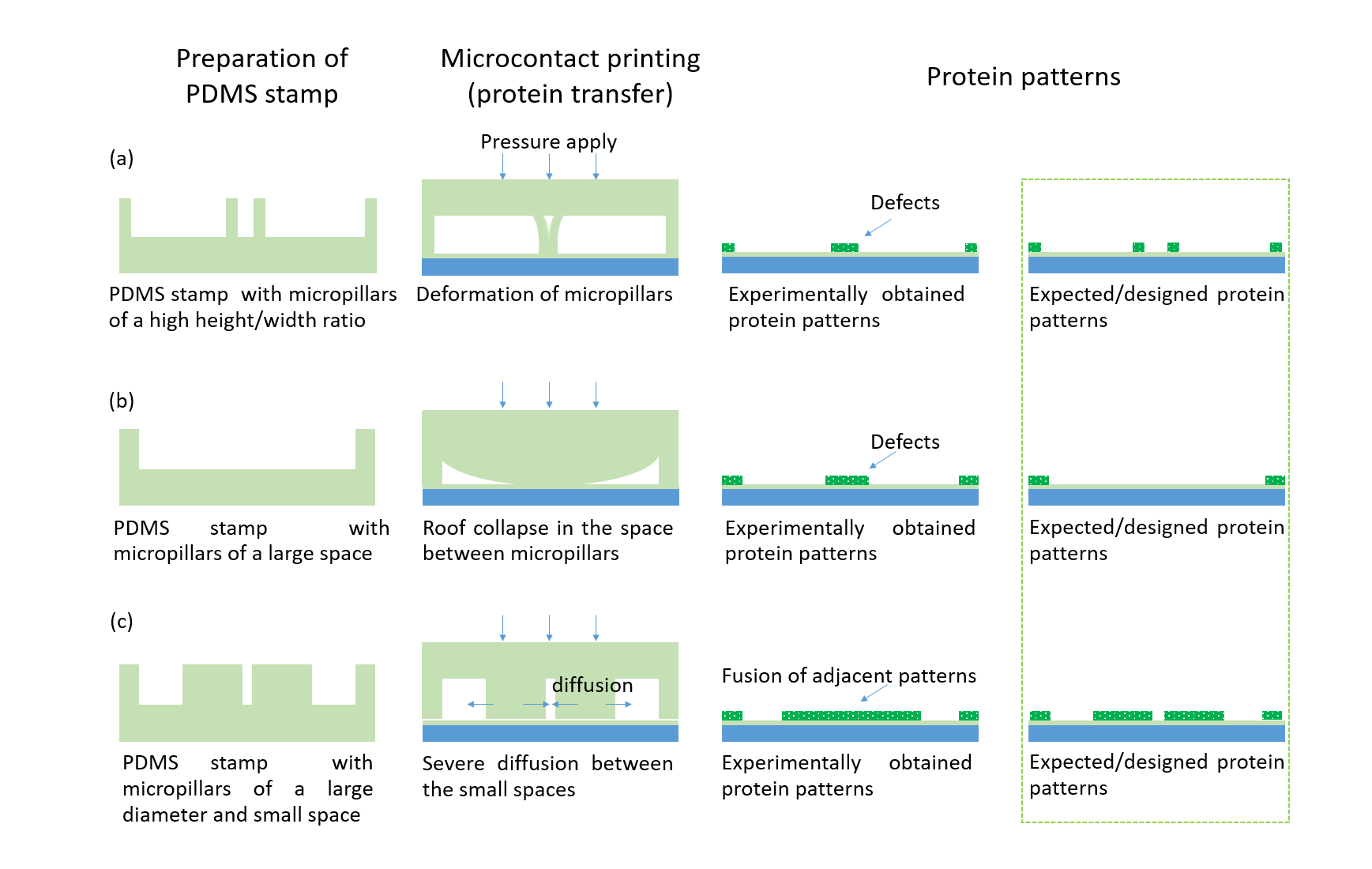


***Fig. S1 Schematic illustration of problems identified in the microcontact printing technique.*** *(a) deformation of micropillars, (b) roof collapse in the space between micropillars, and (c) diffusion between the small spaces between micropillars.*

***Fig. S2 Protein and cell patterns on curved substrates.*** *Representative fluorescence images of protein patterns (a-b) and correspondingly cell patterns (c-d) obtained on curved substrates with various radius of curvature (r=0.5 mm, 1.5 mm, 2.5 mm and 3 mm) in views of different angles. Scale bar: 100 μm.*

**
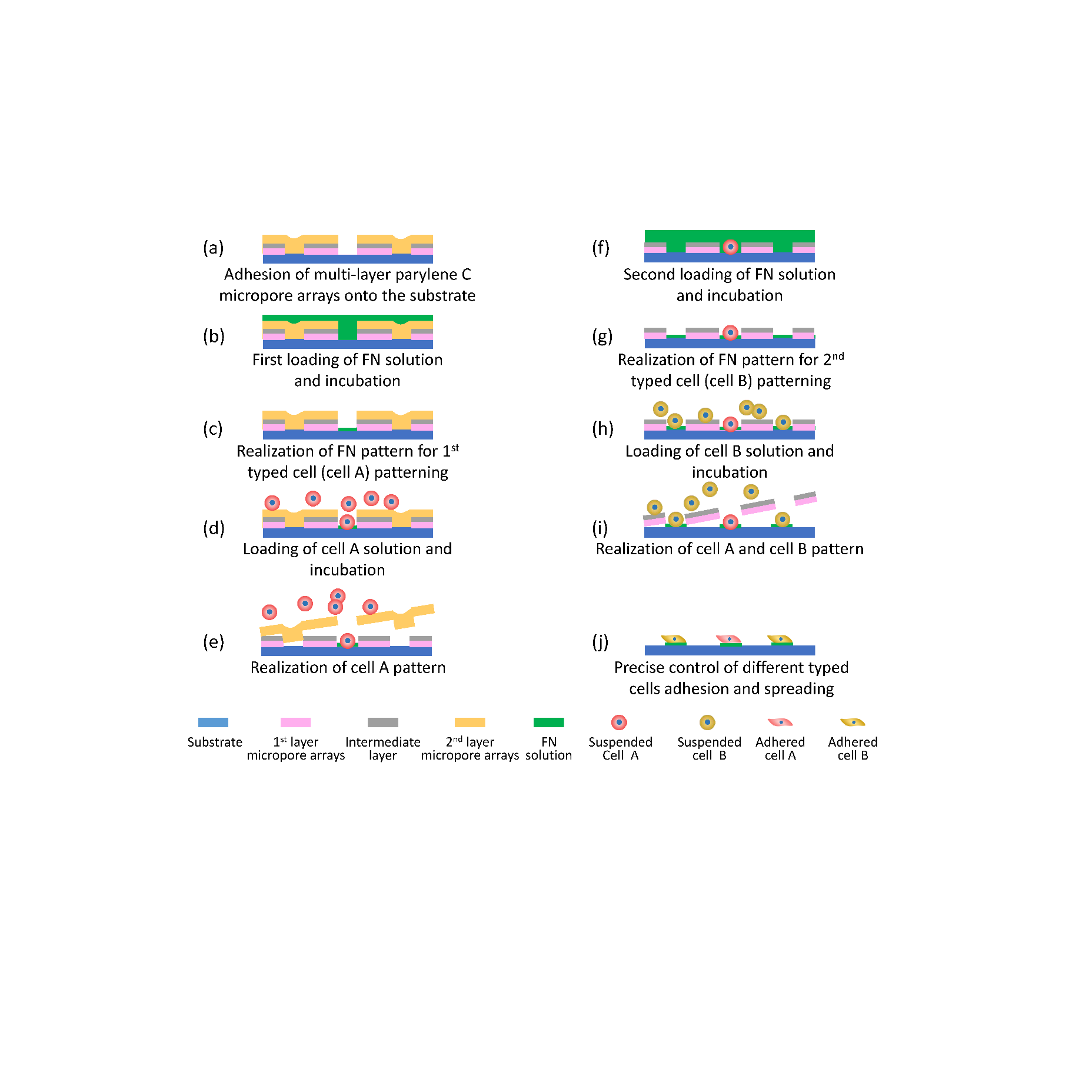
**

***Fig. S3 Schematic illustration of the patterning/alignment of two different typed cells via the multistep solid lift-off processes with multilayer Parylene C micropore arrays as the shadow masks.***
